# Xylanase/β-Glucanase Synergy: Enhancing Dough Structure and Bread Quality in Highland Barley–Wheat Blend

**DOI:** 10.3390/foods15030486

**Published:** 2026-02-01

**Authors:** Menglu Zong, Jiaqi Wang, Tong Wu, Wenjing Ma, Ji Kang, Jinpeng Wang

**Affiliations:** 1 College of Food Science and Engineering, Tianjin University of Science & Technology, Tianjin 300457, China; zong09meng06lu@163.com (M.Z.); m15511206991@163.com (J.W.); 18526321596@163.com (T.W.); 15231119396@163.com (W.M.); 2 Key Laboratory of Geriatric Nutrition and Health, Beijing Technology and Business University, Ministry of Education, Beijing 100048, China; 3 School of Food and Health, Beijing Technology and Business University, Beijing 100048, China

**Keywords:** composite flour, dual-enzyme treatment, non-starch polysaccharides, whole grain dough, whole grain bread

## Abstract

Highland barley (HB), a nutrient-rich grain, is limited in bread applications due to its weak gluten network and high content of non-starch polysaccharides (NSPs) such as β-glucan and arabinoxylan. This study aimed to improve the dough properties and bread quality of a composite flour containing 40% whole-grain highland barley through synergistic use of xylanase and β-glucanase. Rheological analysis revealed that dual-enzyme treatment significantly reduced dough rigidity (G′ decreased by ~40%) and increased extensibility (tan δ raised by ~25%), shifting the network from a brittle NSP-dominated gel toward an elastic gluten-based structure. Low-field NMR showed that enzymes promoted redistribution of water from tightly bound states with NSPs to protein phases, enhancing gluten hydration. Microstructural observations confirmed a more continuous and uniform gluten network with finely embedded starch granules. Consequently, enzyme-treated bread exhibited a 35% higher specific volume, reduced hardness (~50% lower), improved springiness and cohesiveness, and superior sensory scores in texture, taste, and overall acceptability compared to the untreated composite. Single-enzyme treatments yielded partial improvements, highlighting the necessity of synergistic action. These results demonstrate that combined xylanase and β-glucanase treatment effectively mitigates the negative impact of NSPs, enabling the production of high-quality, sensorially appealing HB-enriched bread with optimized structural and textural properties.

## 1. Introduction

The global consumption of bread continues to grow, alongside rising consumer demand for healthy functional foods, driving intensified efforts to enhance the nutritional and functional properties of bread [1]. As a representative whole-grain product, whole wheat bread holds substantial potential for global promotion [2]; however, breads containing whole grains often face challenges such as denser texture, reduced volume, and stronger flavors, which can hinder their large-scale production and consumer acceptance [3].

Highland barley (HB), cultivated in high-altitude regions of China such as Qinghai and Xizang, possesses unique nutritional and health benefits attributed to its distinctive growing environment. Its rising popularity in recent years underscores its potential as a functional food [4]. HB is rich in bioactive compounds such as dietary fiber (predominantly β-glucan and arabinoxylan), high-quality protein (with balanced amino acids), B vitamins, minerals, and polyphenols [5]. These components confer health-promoting effects (e.g., hypoglycemia, lipid regulation, antioxidant, and anti-inflammatory activities) when incorporated into bread [6,7].

Despite its advantages, HB poses critical challenges for yeast-leavened bakery product development. Unlike wheat, HB is dominated by globulin and albumin, with low levels of glutenin and hordein, impeding the formation of a robust gluten network-an essential structure determining dough rheological properties (e.g., elasticity, extensibility, and gas retention) [8,9]. Additionally, HB’s high β-glucan content forms gels during processing, disrupting gluten’s primary and secondary structures, while arabinoxylan (though less abundant) modulates dough viscosity and stability; collectively, these fac-tors compromise gluten network formation, leading to inconsistent quality and struc-tural fragility in HB-containing breads [10,11]. Currently, HB is primarily used in non-fermented foods, and while low β-glucan concentrations can modestly enhance gluten properties [12], structural instability during processing remains a major barrier to its broader application in baked goods.

Industrial attempts to address these issues typically involve blending HB flour with high-gluten wheat flour or wheat gluten to maintain processability and sensory quality [8]. However, enzyme-based strategies have emerged as promising, natural alternatives to chemical additives (e.g., emulsifiers), offering eco-friendly and safe avenues to improve dough rheology, gas retention, and product shelf life [13]. Xylanase, accounting for approximately 40% of baking enzymes, enhances dough relaxation, bread volume, and crumb softness [14], while β-glucanase (BGS) degrades high-molecular-weight β-glucan into oligosaccharides, facilitating gluten cross-linking and reinforcing the gluten network in whole-grain product [15].

Notably, while the individual effects of xylanase and BGS have been explored, research on their synergistic application, particularly in HB based systems, remains limited [16,17]. Combined enzyme treatments are hypothesized to outperform single-enzyme strategies in optimizing product quality. Thus, this study investigated the synergistic effects of xylanase and BGS on the properties of dough and bread produced from a composite flour (40:40% highland barley with bran and 60% high-gluten wheat flour), and used highland barley with bran (HB) and high-gluten wheat flour (WF) as controls. This study aimed to evaluate enzyme-assisted improvement in whole-grain baked products by assessing both dough performance and bread quality, including specific volume, texture, and sensory acceptability, thereby supporting the development of nutritious HB-based products.

## 2. Materials and Methodology

### 2.1. Materials

Whole grain Highland barley (processed from bran-containing highland barley and used as received, without any specific debittering treatment, protein 11.23%, starch 60.13%, moisture 10.71%, ash 1.92%, dietary fiber 10.82%) was obtained from Qinghai–Tibet Tribe Agriculture and Animal Husbandry Development Co., Ltd. (Hainan Tibetan Autonomous Prefecture, Qinghai, China). High-gluten wheat flour (protein 23%, starch 71.25%, moisture 13.02%, ash 0.49%, gluten 31.59%) was purchased from Yihai Kerry Arawana Holdings Co., Ltd. (Shanghai, China). Both types of flour were sieved through an 80-mesh screen. β-Glucanase (activity: 3300 U/mg) was provided by Beijing Solarbio Science & Technology Co., Ltd. (Beijing, China). Xylanase (activity: 300,000 U/g)was provided by Shanghai Yuan Ye Biotechnology Co., Ltd. (Shanghai, China). Additional ingredients included refined granulated sugar (Sukaman brand), unsalted butter (Anchor brand), table salt (China Salt Industry Co., Ltd., Beijing, China), and high-sugar-tolerant instant yeast (Angel Yeast Co., Ltd., YiChang, Hubei, China).

### 2.2. Dough Preparation

The recipe for the composite dough (40) includes: 60% WF, 40% HB, and an appropriate amount of pure water (calculated based on the water absorption rate measured by the powder weight method, at 92%). Additionally, 0.2% of the dual-enzyme preparation was incorporated into three dough formulations, with the activity ratio of β-glucanase to xylanase set at 3:1. In parallel, two corresponding single-enzyme treatments were prepared by adding β-glucanase or xylanase alone to produce unleavened doughs (without a leavening agent). The mixtures were then mixed for 5 min using a dough mixer (ESC1510, Midea, Wuhu, China). The combination mode of this enzyme, the dosage used and the formula proportion within the 40 system were all selected based on the optimal results from preliminary tests and references, with the aim of achieving the best bread quality [18]. Meanwhile, the untreated 40 composite dough, WF and HB were prepared as control groups.

### 2.3. Bread Preparation

The (40) bread was prepared according to the following formulation (per dough piece, ingredient quantities are expressed as a percentage of the total flour weight): 60% WF, 40% HB. Sugar 12%, salt 0.8%, butter 10%, instant yeast 5%, water (added based on farinograph water absorption), and enzymes as described in Section 2.1 [19]. The dry ingredients (excluding yeast) were first homogenized. Yeast and enzyme(s) were then dissolved/dispersed in the water and added to the dry mixture. The dough was mixed for 5 min in a dough mixer, after which butter was incorporated and mixing continued for an additional 5 min. The dough was placed in a baking pan, covered with plastic film, and proofed at 38 °C for 60 min. Finally, the proofed dough was baked at 180 °C for 12 min.

### 2.4. Dough Dynamic Rheological Measurements

Dynamic rheological properties of the dough were measured using a rheometer (MARS 60, Thermo Fisher Scientific, Karlsruhe, Germany), following the method reported by Wang et al. [20] with minor adaptations. Briefly, approximately 4 g of dough was placed between parallel plates (35 mm diameter, 1 mm gap) and sealed around the edges with silicone oil to minimize moisture loss. Samples were rested for 5 min to release residual stress prior to measurement. Frequency sweep tests were performed at a fixed strain of 0.1% and a temperature of 25 °C, with frequency varying from 0.1 to 10 Hz. The storage modulus (G′), loss modulus (G″) and tan δ (G′/G″) of the dough were recorded.

### 2.5. Dough Creep Recovery Scan

The creep–recovery properties of the dough were measured using a rheometer (MARS 60, Thermo Scientific, Germany) according to the method described by Gong et al. [21]. The test was performed under a constant stress of 50 Pa at 25 °C. Creep deformation was recorded for 300 s, after which the stress was removed and the recovery phase was monitored for an additional 300 s.

### 2.6. Dough Texture Profile Analysis (TPA)

Highland barley dough samples were prepared as cylinders (2 cm height × 1 cm diameter) and subjected to texture analysis using a texture analyzer (TA.XTC, Baosheng, Shanghai, China) equipped with a P/36R probe. The test was conducted under the following settings: pre-test speed, 2 mm/s; test speed, 1 mm/s; post-test speed, 2 mm/s; trigger force, 5 g; and interval time, 2 s [8]. Hardness, springiness, chewiness, cohesiveness and gumminess were calculated from the TPA curve. TPA measurements were obtained in gram-force (gf), while presenting the units simply as grams (g) for the relevant parameters.

### 2.7. Dough Water Migration Analysis by Low-Field Nuclear Magnetic Resonance (LF-NMR)

The moisture distribution in the dough was analyzed using low-field nuclear magnetic resonance (LF-NMR) (MicroMR-25, Niumag, Suzhou, China). Samples weighing 5 g were wrapped in cling film and placed into an NMR tube. The following pulse sequence parameters were applied: repetition time (TW) = 2000 ms, number of scans (NS) = 2, 90° pulse width (P2) = 13.04 μs, echo time (TE) = 0.5 ms, and number of echoes (NECH) = 18,000.

### 2.8. Dough Fourier Transform Infrared Spectroscopy (FTIR) Analysis

The infrared spectra of the samples were acquired using a Fourier transform infrared (FT-IR) spectrometer (Model IS50, Thermo Nicolet Corporation, Washington, DC, USA), following the procedure reported by Xin et al. [22]. Approximately 1 mg of each sample was finely mixed with 150 mg of pre-dried potassium bromide (KBr), ground thoroughly, and then pressed into transparent pellets. Spectral data were collected over the wavenumber range of 400–4000 cm^−1^ with 32 scans per sample, and processed using Omnic software (version 8.2, Thermo Scientific, Madison, WI, USA).

### 2.9. Dough Microstructural Observation by Scanning Electron Microscopy (SEM)

Following freeze-drying, dough samples were fixed onto circular specimen stubs using conductive adhesive. The samples were then evacuated in a vacuum chamber and sputter-coated with a thin gold layer. Microstructural images were acquired using a scanning electron microscope (SU1510, Hitachi, Tokyo, Japan) operated at an acceleration voltage of 15 kV at a magnification of ×1500 [23].

### 2.10. Bread Specific Volume Determination

The specific volume of bread was determined as the ratio of volume (mL) to weight (g). After baking, bread samples were cooled at room temperature for 1 h prior to volume measurement using a millet displacement method. In this procedure, a graduated cylinder was first filled with a known volume of millet. The millet was then removed, the bread sample was placed into the cylinder, and the millet was poured back. The total volume was recorded, and the bread volume was calculated by subtracting the initial millet volume from the total displacement volume.

### 2.11. Bread Texture Profile Analysis (TPA)

Bread samples were cut into cubes (1.5 × 1.5 × 1.5 cm^3^) for texture profile analysis (TPA) using a texture analyzer (TA.XTC, Baosheng, Shanghai, China) equipped with a P/36R cylindrical probe. The TPA settings were as follows: pre-test speed, 2 mm/s; test speed, 1 mm/s; post-test speed, 2 mm/s; and a 5 s interval between two consecutive compression cycles. Three replicate measurements were performed for each sample [24]. TPA measurements were obtained in gram force, while presenting the units simply as grams (g) for the relevant parameters.

### 2.12. Bread Sensory Evaluation

Sensory evaluation of the freshly baked bread was conducted after cooling to room temperature. Twenty-two untrained panelists participated in a consumer preference test using a 9-point hedonic scale (1 = dislike extremely, 5 = neither like nor dislike, 9 = like extremely). The panelists rated each sample for appearance, color, aroma, texture, and overall acceptability. To minimize potential bias, bread samples were presented to the participants in randomized order.

### 2.13. Statistical Analysis

All measurements were conducted using three independently prepared samples per group (n = 3). All data are expressed as mean ± standard deviation. Statistical analysis was performed using SPSS 27.0 software (SPSS Inc., Chicago, IL, USA), with significant differences within groups determined by one-way analysis of variance (ANOVA) followed by Duncan’s multiple range test (*p* < 0.05). Means within the same group labeled with different lowercase letters are significantly different. Figures were generated using Origin 2024 software (Origin Lab, Northampton, MA, USA) and Graphpad Prism (version 8.0, Graphpad Software, LaJolla, CA, USA).

## 3. Results and Analysis

### 3.1. Dynamic Rheological Properties

**Figure 1:**
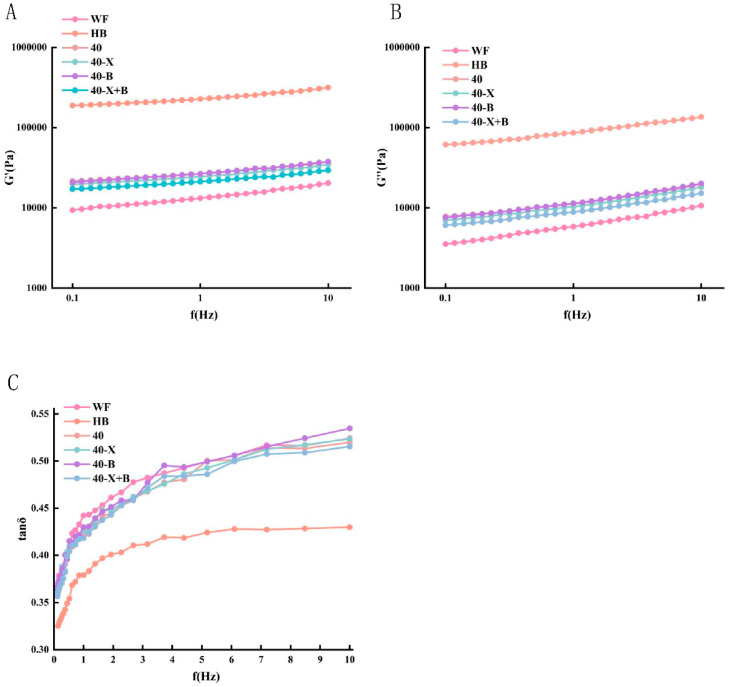
The effects of various enzyme treatments on G′ (**A**), G″ (**B**), and tan δ (**C**) of the dough. WF: completely made from high-gluten wheat flour, HB: completely made from highland barley with bran, 40: composed of 40% HB and 60% WF, 40-X: sample 40 was treated with xylanase, 40-B: sample 40 was treated with β-glucanase, 40-X+B: sample 40 was treated with β-glucanase.

The viscoelastic properties of dough, which are essential for predicting its processability and final product quality [25], are largely determined by the gluten network. This network enables the dough to retain gas and achieve appropriate expansion during baking [26,27]. The loss factor (tan δ), defined as the ratio of loss modulus (G″) to storage modulus (G′), reflects the viscoelastic balance of the dough, with lower values indicating more dominant elastic (solid-like) behavior [28]. As shown in Figure 1, all dough samples exhibited G′ values consistently higher than G″ across the tested frequency range, with tan δ remaining below 1, confirming their solid-like, gel-type character [29]. Among them, the pure highland barley (HB) dough displayed the highest G′ and G″ values (Figure 1A,B), indicating a rigid and dense network. This rigidity is attributed to the high content of non-starch polysaccharides (NSPs), primarily β-glucan and arabinoxylan, which form a tightly packed gel structure with limited extensibility. The correspondingly low tan δ value (Figure 1C) further confirms the predominance of elasticity and restricted viscous dissipation, resulting in a hard, brittle texture. This is consistent with the results of studies such as Ying et al., which show that substrates rich in β-glucan/arabinogalactan can enhance the water absorption capacity and increase the apparent hardness of cereal dough, thereby increasing the modulus and limiting the deformation under oscillatory shear [30].

In contrast, the pure high-gluten wheat flour (WF) dough showed the lowest G′ and G″ values, reflecting a homogeneous, elastic, and highly extensible gluten network. Its higher tan δ indicates greater viscous fluidity, consistent with the flexible and mobile nature of a well-developed gluten matrix.

The introduction of WF into the HB system (composite 40) substantially reduced both moduli and increased tan δ, demonstrating that wheat gluten effectively established a continuous protein framework. This shift mitigated the excessive rigidity imposed by HB-derived NSPs and transitioned the dominant network from a brittle NSP gel to a more elastic gluten structure, thereby creating a necessary foundation for enzymatic modification.

Within this composite system, the combined application of β-glucanase and xylanase further reduced G′ and G″ while increasing tan δ, indicating that the dual-enzyme treatment synergistically hydrolyzed β-glucan and arabinoxylan. This process effectively eliminated the physical interference of NSPs with the gluten network, markedly lowering dough rigidity, enhancing network fluidity, and improving extensibility. Importantly, the resulting moduli remained higher than those of pure WF, indicating that the optimized network retained sufficient structural integrity. This is consistent with the research results of Liu et al. [17,31].

This rheological study confirms that the synergistic action of xylanase and β-glucanase did not disrupt the dough network. Instead, it precisely modulated the overly rigid HB-dominated system toward an optimal viscoelastic balance between the excessively soft WF and the excessively stiff pure HB. This adjustment significantly improved dough handling properties, yielding a structure that is simultaneously softer, more extensible, and adequately strong—a finding consistent with earlier reports [27].

### 3.2. Creep-Recovery Test

**Figure 2:**
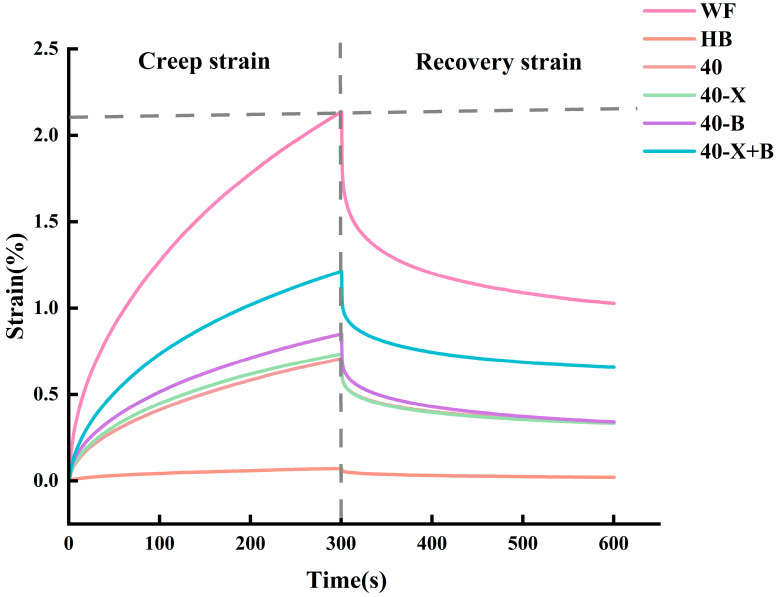
The creep–recovery curves of doughs subjected to different enzyme treatments. WF: completely made from high-gluten wheat flour, HB: completely made from highland barley with bran, 40: composed of 40% HB and 60% WF, 40-X: sample 40 was treated with xylanase, 40-B: sample 40 was treated with β-glucanase, 40-X+B: sample 40 was treated with β-glucanase.

The creep-recovery test, which evaluates dough deformation under constant stress and its subsequent recovery after stress removal [32]. It revealed distinct viscoelastic behaviors across samples, with dual-enzyme treatment exerting a pronounced influence on creep properties. Creep compliance at the end of the loading phase (Jmax) reflects dough stiffness: higher Jmax indicates greater deformability, which correlates with improved extensibility and softer texture [33,34]. As shown in Figure 2, different doughs exhibited markedly different structural responses under sustained stress. Contrary to the conventional view that high creep strain signifies poor quality, the present study demonstrates that elevated strain can instead reflect a network’s enhanced resilience and energy-absorbing capacity.

The WF dough showed the highest Jmax, indicating maximum deformation under stress. This should not be interpreted as structural weakness but rather as a hallmark of its high-quality gluten network, which allows reversible molecular-chain sliding and rearrangement under load, followed by efficient entropy-driven recovery upon stress release. In contrast, the HB dough displayed the lowest strain, reflecting not high strength but pronounced brittleness and poor ductility. Its gel-like network, dominated by non-starch polysaccharides (NSPs) such as β-glucan and arabinoxylan, exhibits limited chain mobility and undergoes irreversible fracture once the yield point is reached, resulting in a macroscopically “hard and brittle” material prone to collapse during fermentation. This structural constraint is also reflected in creep behavior: when water mobility is restricted and NSP-associated viscosity competes with gluten development, creep compliance decreases and recoverable strain is reduced, ultimately increasing resistance to deformation and lowering gas-holding tolerance [35,36].

Incorporating high-gluten wheat flour into the HB system (composite 40) markedly increased Jmax, surpassing that of pure HB. This shift indicates that wheat gluten provides the continuous elastic framework lacking in highland barley, transforming the dough from a brittle material into one with fundamental extensibility and deformability—a critical prerequisite for further enzymatic improvement.

Single-enzyme treatments (40-X and 40-B) produced curves similar to the untreated composite 40, suggesting that neither xylanase nor β-glucanase alone substantially alters the network structure. The slightly better performance of 40-B may be attributed to the predominant role of β-glucan as the main resistant component in the highland barley system.

Notably, the dual-enzyme treatment (40-X+B) induced a pronounced upward shift in the creep curve, bringing it close to the ideal profile of WF. This key finding demonstrates that xylanase and β-glucanase act synergistically to hydrolyze arabinoxylan and β-glucan, effectively dismantling the rigid NSP framework. This process liberates water-holding space, enables gluten proteins to fully extend and cross-link, and ultimately establishes a resilient, wheat-like network that combines high extensibility with excellent recovery.

Thus, in this context, “high strain” reflects not structural failure but remarkable extensibility and toughness. The dual-enzyme treatment fundamentally transformed the composite system from the “low strain–poor recovery” mode characteristic of HB to a “high strain–high recovery” mode resembling WF. This transition provides the structural foundation for improved fermentation tolerance and baking potential [37].

### 3.3. Evaluation of Dough Texture Characteristics

The texture profile analysis (TPA) results are summarized in Table 1. Both the addition of wheat flour (WF) and enzyme treatment systematically influenced the textural properties of the composite doughs.

The highland barley (HB) dough exhibited a failure pattern characterized by high hardness, low elasticity, and poor cohesiveness. Its exceptionally high adhesiveness combined with very low chewiness indicates the formation of a dense yet brittle gel network lacking structural toughness. This network offers high initial resistance to compression but undergoes rapid failure under sustained deformation due to restricted molecular chain mobility, resulting in inefficient mechanical energy dissipation.

In the composite dough (40), hardness and adhesiveness were significantly reduced compared to HB, confirming the preliminary formation of a gluten network. However, cohesiveness and elasticity did not improve significantly, suggesting that network development remained incomplete. This is primarily attributed to the abundant non-starch polysaccharides (NSPs) in the system, which impose steric hindrance and compete for water, thereby restricting gluten protein extension and interaction [38].

Enzyme treatment markedly enhanced dough cohesiveness and elasticity, reaching levels comparable to those of WF. This improvement results from the efficient degradation of NSPs, which removes physical barriers to protein network formation, promotes adequate hydration, and facilitates gluten cross-linking, ultimately yielding a continuous, stable, and finely structured three-dimensional network [39].

Notably, the dual-enzyme group (40-X+B) achieved high cohesiveness and elasticity while maintaining hardness and adhesiveness values similar to WF. The moderate increase in chewiness reflects an optimized balance between energy dissipation and structural recovery, corresponding to an improved chewing experience. In addition, the TPA trends in Table 1 are consistent with the creep–recovery response discussed in Section 3.2 (Figure 2). Doughs with improved deformability and recovery under sustained stress generally exhibited lower hardness and higher cohesiveness/elasticity, supporting a more continuous and resilient network structure after enzymatic treatment.

### 3.4. Determination of Moisture Distribution in Dough

Low-field nuclear magnetic resonance (LF-NMR) was employed to analyze the mobility states of water in the dough systems. The signal amplitude on the y-axis reflects proton density, while the peak area corresponds to the relative water content in each mobility population [40]. Water status critically influences dough plasticity and network development, with shorter T_2_ relaxation times indicating water more tightly bound to the matrix (proteins and polysaccharides) and thus possessing lower mobility.

**Figure 3:**
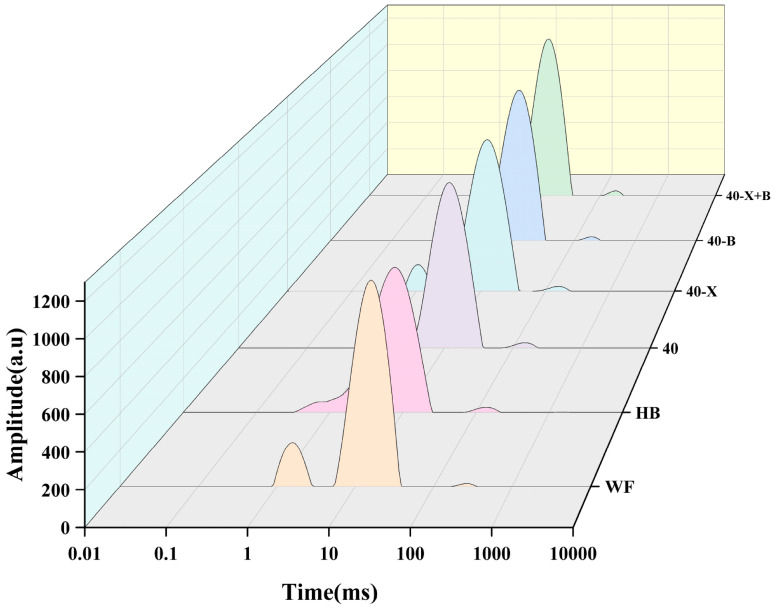
Water distribution in different treated doughs. WF: completely made from high-gluten wheat flour, HB: completely made from highland barley with bran, 40: composed of 40% HB and 60% WF, 40-X: sample 40 was treated with xylanase, 40-B: sample 40 was treated with β-glucanase, 40-X+B: sample 40 was treated with β-glucanase.

As shown in Figure 3, the main peak of the highland barley (HB) dough shifted markedly toward shorter T_2_ times, accompanied by increased signal intensity. This indicates strong interactions between water and the non-starch polysaccharide (NSP) gel network, leading to a high proportion of tightly bound water [41]. In this state, water is largely immobilized by NSP components, which not only impedes proper hydration of gluten proteins but also reduces overall molecular mobility, resulting macroscopically in a brittle, rigid structure with limited elastic recovery [8].

In contrast, the wheat flour (WF) dough exhibited a broader relaxation spectrum, reflecting a more diverse distribution of water phases—including water tightly associated with gluten and starch, capillary water within the network interstices, and a portion of free water [42,43]. This polymorphic water profile supports the formation of a uniform, elastic gluten network: fully hydrated proteins adopt relaxed conformations to build a continuous framework, while weakly bound and free water act as plasticizers, conferring essential viscoelasticity and deformability [44].

The composite dough (40) showed a water distribution intermediate between HB and WF, though closer to HB. This reduction in overall water mobility highlights the need for further optimization.

Both single-enzyme treatments (40-X and 40-B) enhanced the signal in the short-T_2_ region while reducing the signal at longer T_2_ times, confirming that xylanase and β-glucanase each release bound water via specific hydrolysis of their respective NSP substrates [45]. This shifts water into forms more tightly associated with proteins and other matrix components, thereby preliminarily adjusting water availability. The subtle differences in signal enhancement between the two enzymes reflect their distinct impacts on strongly bound water pools [46].

Most notably, the dual-enzyme treatment (40-X+B) produced the strongest signal in the shortest T_2_ region. This demonstrates that xylanase and β-glucanase act synergistically to efficiently degrade NSPs, not only liberating water previously trapped by NSPs but, more importantly, redirecting it toward the gluten protein phase. This redistribution promotes the formation of a highly hydrated, dense protein network, ultimately yielding a system with a maximized proportion of tightly bound water.

Through this deep water rearrangement, the dual-enzyme system fundamentally alters water functionality: water shifts from a state that constrains network extensibility (tightly bound to NSPs) to one that enhances gluten elasticity (effectively plasticizing the protein network). This transformation underpins a material transition from a brittle, hydrogen-bond-dominated gel (typical of HB) toward a high-quality, elastic gluten network (characteristic of WF). The extensive redistribution of water from non-starch polysaccharides (NSP) to the gluten layer, as revealed by low-field nuclear magnetic resonance (LF-NMR) technology, provides a fundamental mechanistic explanation for the observed macroscopic improvements in creep recovery and texture analysis. The water previously fixed by the rigid non-starch polysaccharide gels was released and subsequently absorbed by the gluten proteins, directly facilitating the transformation of the composite dough network structure. Therefore, the LF-NMR data played a crucial bridging role, linking the double-enzyme treatment with the significant improvement in dough rheological properties and the enhancement of texture characteristics.

### 3.5. FTIR Analysis

**Figure 4:**
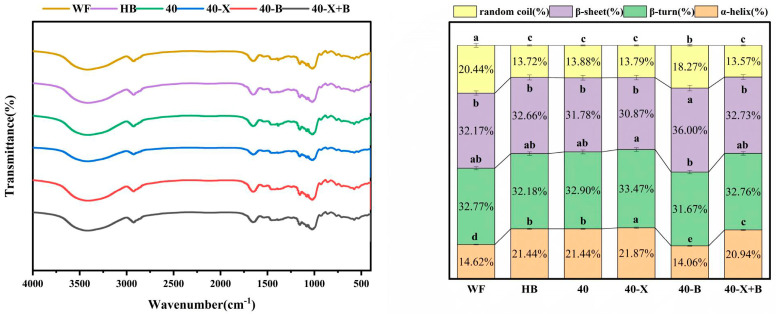
Effects of Various Treatments on the Infrared Spectrum of Dough. WF: completely made from high-gluten wheat flour, HB: completely made from highland barley with bran, 40: composed of 40% HB and 60% WF, 40-X: sample 40 was treated with xylanase, 40-B: sample 40 was treated with β-glucanase, 40-X+B: sample 40 was treated with β-glucanase. ^a–e^ Different letters indicate significant differences between groups (*p* < 0.05).

Fourier transform infrared spectroscopy (FTIR) was used to examine changes in the protein secondary structure of doughs subjected to different treatments [8]. The spectra of the samples are presented in Figure 4. No new absorption peaks appeared, nor did existing peaks disappear in the main characteristic regions, indicating that the treatments did not generate new functional groups and that intermolecular interactions remained predominantly non-covalent, such as hydrogen bonding.

Quantitative analysis of the amide I band, performed by deconvolution and curve fitting, revealed subtle but consistent variations in secondary structure composition among the samples, with the contents of β-sheet (1620–1640 and 1688–1695 cm^−1^), α-helix (1650–1660 cm^−1^), β-turn (1665–1680 cm^−1^), and random coil (1640–1650 cm^−1^) calculated as the ratio of each characteristic peak area to the total amide I band area [47]. Although overall differences were not statistically significant, discernible trends were observed. The wheat flour (WF) dough, which serves as a reference for an optimal network, was rich in β-sheets (32.77%) and β-turns (32.17%). In contrast, highland barley (HB) dough contained a higher intrinsic proportion of α-helices (21.44%).

Notably, treatment 40-B shifted the structural balance, increasing the proportion of β-turns while reducing α-helix content. This change likely stems from the specific hydrolysis of high-viscosity β-glucan by β-glucanase, which abruptly alters the rheological microenvironment and perturbs local protein conformation or intermolecular arrangement.

The most striking result was observed in the dual-enzyme treatment (40-X+B), where the secondary-structure proportions tended to return to a balanced state intermediate between HB and WF, with a β-sheet/β-turn ratio similar to that of the untreated controls. Together with the lack of statistical significance, this suggests that the synergistic enzyme action does not fundamentally reconfigure protein secondary structures at the molecular level, but rather operates through more subtle, system-wide adjustments.

FTIR analysis thus indicates that the marked improvement in the composite highland barley-wheat system is not driven by global chemical restructuring of protein conformations. Instead, it arises from physical reorganization mediated by enzymatic action. Although 40-B alone induced a detectable conformational trend, the dual-enzyme treatment caused no statistically significant change in secondary structure. This implies that the core of the synergy lies in the comprehensive hydrolysis of non-starch polysaccharides (NSPs), which removes physical barriers in the protein network and triggers a system-wide redistribution of water.

By optimizing the physical environment, previously constrained protein molecules are able to extend and cross-link more effectively, utilizing their inherent structural motifs (e.g., β-sheets and β-turns) to assemble a macroscopically superior network—without substantially altering the intrinsic conformation of individual proteins [48].

### 3.6. SEM of Dough

**Figure 5:**
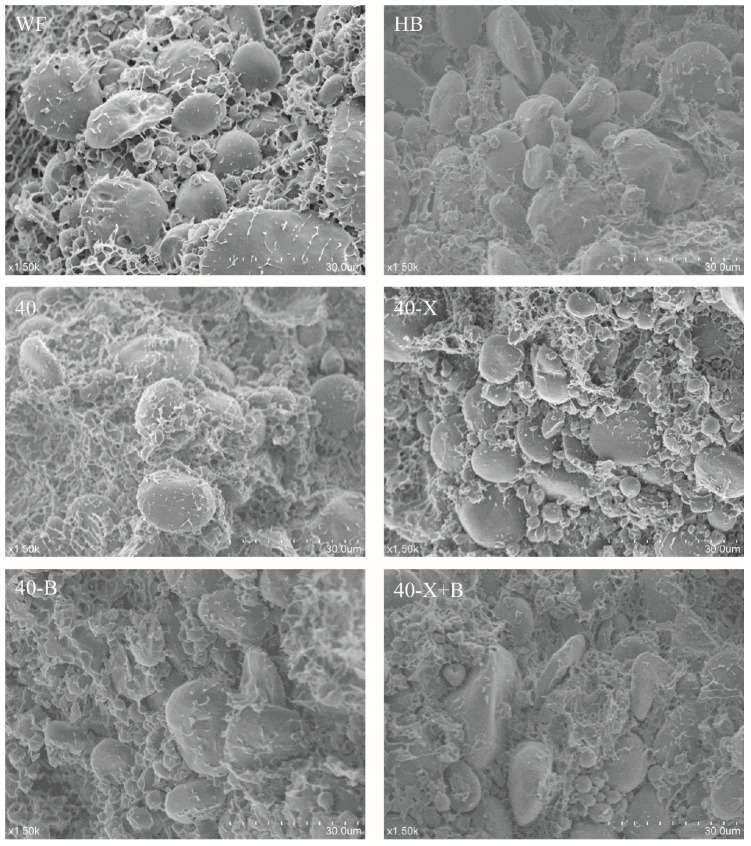
The SEM effects of various treatments on dough. WF: completely made from high-gluten wheat flour, HB: completely made from highland barley with bran, 40: composed of 40% HB and 60% WF, 40-X: sample 40 was treated with xylanase, 40-B: sample 40 was treated with β-glucanase, 40-X+B: sample 40 was treated with β-glucanase.

Scanning electron microscopy (SEM) images of the dough samples are shown in Figure 5. Unlike wheat flour (WF), which is dominated by gliadin and glutenin, highland barley (HB) contains significant amounts of albumin and globulin in addition to hordein and glutenin. Upon hydration, gliadin and glutenin in WF form a strong, elastic gluten network, whereas the more heterogeneous protein composition of HB impedes the development of a cohesive network structure.

Consistent with this distinction, the HB dough displayed a dense, rough, and discontinuous gel-like microstructure, with starch granules largely exposed and lacking a continuous protein film (Figure 5). In contrast, all composite doughs exhibited a continuous gluten-protein matrix that effectively embedded the starch granules, confirming that the addition of high-gluten wheat flour successfully established a foundational network framework.

Compared with the uniform, dense structure of WF, the basic composite dough (40) showed a coarser network, featuring more exposed starch granules and larger, irregularly distributed pores. Enzymatic treatment markedly refined the microstructure, with the most pronounced improvement observed in the dual-enzyme group (40-X+B). Here, the gluten network became more continuous and uniform, starch granules were fully embedded, and the pore structure appeared fine and regular—findings consistent with previous reports [8]. The microstructural refinement observed via SEM is consistent with the molecular-level trends revealed by the FTIR analysis, thereby providing visual support for the spectroscopic interpretation. Specifically, FTIR indicated no statistically significant changes in protein secondary structure after enzymatic treatment, suggesting that the SEM-observed improvements (more continuous network and enhanced starch embedding, particularly in 40-X+B) mainly arise from system-level physical reorganization.

### 3.7. Specific Volume of Bread

**Figure 6:**
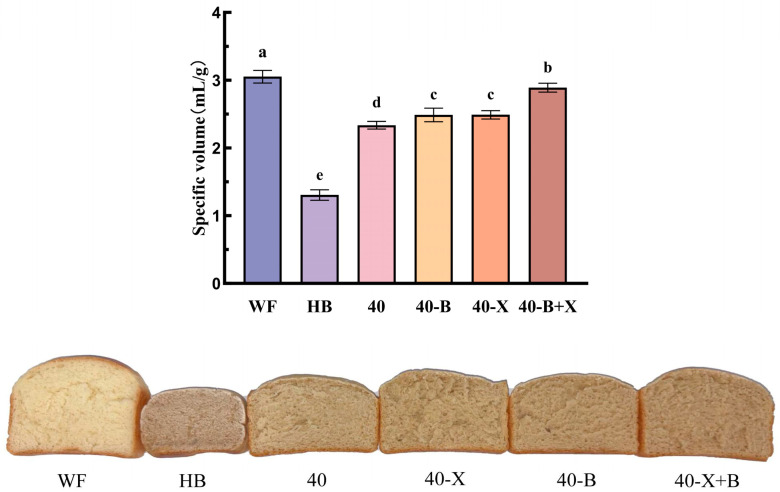
The effects of various treatments on the specific volume and appearance of bread. WF: completely made from high-gluten wheat flour, HB: completely made from highland barley with bran, 40: composed of 40% HB and 60% WF, 40-X: sample 40 was treated with xylanase, 40-B: sample 40 was treated with β-glucanase, 40-X+B: sample 40 was treated with β-glucanase. ^a–e^ Different letters indicate significant differences between groups (*p* < 0.05).

Bread specific volume is a fundamental and comprehensive indicator of baking quality, directly reflecting the dough’s gas-production and gas-retention capacities during fermentation and baking. Consumers generally prefer bread with higher specific volume [49], which is also associated with more porous crumb structure [50]. As shown in Figure 6, significant differences (*p* < 0.05) were observed among treatments, confirming at the product level the conclusions drawn from earlier rheological, textural, and moisture-distribution analyses. The wheat flour (WF) bread displayed the highest specific volume, reflecting the ideal balance of its gluten network between gas retention and structural extensibility. Furthermore, the bread images provided qualitative and intuitive support for the specific volume results. The internal structure of the HB sample’s bread was very compact with limited pore development; while the composite bread and the bread treated with enzymes showed that the internal structure gradually became more loose and the pore distribution became more uniform. Particularly, the internal tissue density of the 40-X+B sample’s bread was lower than that of the 40 sample, which was consistent with its higher specific volum. Overall, the visual appearance was consistent with the quantitative measurement results, indicating that the volume expansion of the enzymatically treated composite bread had improved. This network forms a uniform, strong, and elastic protein matrix that effectively traps carbon dioxide during fermentation and prevents bubble coalescence before starch gelatinization, thereby maximizing loaf volume [51].

In contrast, highland barley (HB) bread showed the lowest specific volume, a consequence of the rigid, brittle, and poorly extensible non-starch polysaccharide (NSP) gel network. This structure exhibits pronounced brittleness and inadequate ductility, which hinder effective gas retention, leading to considerable gas escape, localized collapse, and ultimately limited volume expansion. Similar volume limitations in highland barley formulations have been linked to β-glucan and arabinoxylan-associated viscosity and competitive water binding, which weaken gluten-mediated gas-cell stabilization [50].

The composite dough (40) exhibited significantly greater specific volume than HB, confirming that the addition of high-gluten wheat flour to establish a basic gluten network is essential for improving the volume of highland barley-based bread. Both single-enzyme treatments (40-X and 40-B) improved specific volume relative to the untreated composite, but no significant difference was observed between them. This suggests that in a complex composite system, a single enzyme can only partially modify the network and has limited ability to overcome the gas-retention constraints imposed by NSPs. Recent work on highland barley composite breads similarly reports that enzyme-assisted hydrolysis targeting NSPs can enhance gas retention and loaf expansion, with combined/stronger NSP-modulating strategies generally showing more pronounced baking-performance gains [52].

Notably, the dual-enzyme treatment (40-X+B) achieved the highest specific volume among all highland barley-containing samples, second only to WF, and was significantly superior to both single-enzyme groups. This result provides clear product-level evidence for the synergistic action of xylanase and β-glucanase. By more completely degrading NSP components, the enzymes promote water redistribution and enable full development of the gluten network, substantially enhancing the dough’s ability to produce and retain gas during baking. The increased bread volume after enzymatic treatment can be attributed to the structural and rheological improvements observed in the dough. Specifically, reduced dough hardness and enhanced extensibility favor gas retention and expansion during fermentation and baking. In addition, the redistribution of water from NSP-associated bound states toward the gluten phase likely facilitates gluten hydration and network development, which is reflected by a more continuous and uniform microstructure. Collectively, these changes support the formation of a stable, elastic matrix that retains CO_2_ and expands more uniformly, resulting in higher loaf volume and a finer crumb structure.

### 3.8. Bread Texture

The textural properties of bread are key quality indicators. As presented in Table 2, significant changes were observed in hardness, springiness, chewiness, cohesiveness, and gumminess, which aligned closely with the earlier rheological and moisture-distribution results.

Bread hardness is generally inversely related to quality [53]. The highland barley (HB) bread showed the highest hardness, consistent with its elevated storage modulus (G′) in rheology and poor strain recovery in creep tests, confirming a rigid network dominated by non-starch polysaccharides (NSPs). The composite (40) exhibited markedly lower hardness, indicating that the gluten network substantially improved the system’s mechanical properties. This reduction in hardness can be directly traced to the structural softening observed in dough rheology, where enzyme treatments significantly lowered G′ and increased tan δ, indicating a shift from a brittle NSP-dominated gel toward a more elastic gluten-based network. Furthermore, the enhanced extensibility and recovery capacity demonstrated in creep-recovery tests foreshadowed the improved mechanical tolerance of the dough during baking, contributing to a softer crumb structure.

While enzyme treatments (40-X, 40-B, and 40-X+B) further reduced hardness, no significant differences were found among them. This suggests a threshold effect in hardness improvement, with single-enzyme treatments already achieving most of the possible reduction. This is consistent with the research results of Hu et al. [54].

Springiness and cohesiveness reflect fundamental network integrity. The low values for HB confirm its brittle, discontinuous structure. The recovery of springiness in composite 40 to near-WF levels demonstrates that gluten-network formation is essential for elastic recovery. Interestingly, the dual-enzyme treatment (40-X+B) did not show superior springiness compared to single-enzyme treatments and was even slightly lower, possibly due to excessive polysaccharide degradation that altered key elastic structural units [55].

Chewiness and gumminess, as integrated texture parameters, better reflect the actual eating experience. The chewiness of (40-X+B) was not significantly different from WF, while its gumminess was significantly lower than HB. This indicates an optimized texture profile—balanced chew strength with reduced stickiness—which corresponds to the improved extensibility and recovery observed in creep-recovery tests. Thus, dual-enzyme treatment achieves comprehensive texture enhancement through structural refinement of the dough network. The texture profile of the final bread is a direct outcome of the sequential structural modifications induced by enzymatic treatment. The reduction in hardness correlates with decreased dough rigidity and improved extensibility; the enhanced springiness and cohesiveness are consistent with optimized water distribution and a continuous gluten network; and the balanced chewiness and gumminess reflect the overall network resilience and recovery capacity. This multi-dimensional coordination effect, ranging from the rheological properties of the dough, the dynamic characteristics of water, to the microstructure and the final product’s texture, has confirmed that the synergistic action of xylanase and β-glucanase fundamentally transforms the composite bread (40) into a high-quality baked product.

### 3.9. Sensory Evaluation of Bread

**Figure 7:**
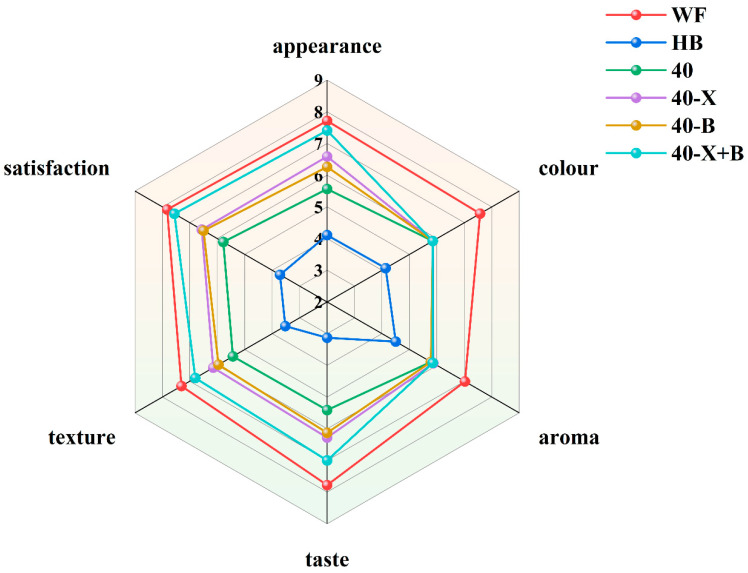
Sensory Evaluation of Breads. WF: completely made from high-gluten wheat flour, HB: completely made from highland barley with bran, 40: composed of 40% HB and 60% WF, 40-X: sample 40 was treated with xylanase, 40-B: sample 40 was treated with β-glucanase, 40-X+B: sample 40 was treated with β-glucanase. 1 = dislike extremely, 2 = dislike very much, 3 = moderately dislike, 4 = slightly dislike, 5 = neither like nor dislike, 6 = slightly like, 7 = moderately like, 8 = like very much, 9 = like extremely.

The sensory attributes of the bread samples were evaluated using a 9-point hedonic scale, and the results are summarized in Figure 7. Panelists assessed appearance, color, aroma, taste, texture, and overall acceptability.

Wheat flour (WF) bread received the highest scores for taste and overall acceptance, favored for its balanced flavor and refined texture. In contrast, highland barley (HB) bread scored significantly lower, especially in taste and texture, consistent with its previously measured high hardness and gumminess.

Enzyme-treated samples showed a clear gradient of improvement. Single-enzyme treatments (40-X and 40-B) notably enhanced key sensory indicators, while the dual-enzyme treatment (40-X+B) achieved the best results in taste, texture, and overall acceptability, bringing its sensory profile closest to that of WF bread. Notably, this sample also received high appearance scores, with its uniform and fine crumb structure widely praised by panelists—a finding consistent with reports that xylanase can improve bread quality [56].

It should be noted, however, that enzyme treatments had limited impact on color and aroma. Scores for these two attributes remained relatively stable and were significantly lower in all highland barley-containing samples compared to WF, indicating that raw-material properties remain the dominant factors for these characteristics.

## 4. Conclusions

This study demonstrates that the synergistic application of xylanase and β-glucanase effectively overcomes the key technological limitations of highland barley (HB) in bread-making—namely its weak gluten network and high content of non-starch polysaccharides (NSPs). The incorporation of high-gluten wheat flour provided an essential protein framework, while the dual-enzyme system acted as a precise “structural modulator” that hydrolyzed β-glucan and arabinoxylan, liberated bound water, and promoted gluten hydration and cross-linking. The dual-enzyme treatment technology can optimize the rheological properties of the dough, enhance its extensibility and recovery ability, and promote the formation of a continuous gluten network. This network can encapsulate the starch particles, thereby improving the quality of the bread. Compared with ordinary bread, its bulk density has increased by approximately 35%; its hardness has decreased by about 50%; and its sensory characteristics are very similar to those of wheat bread. While single-enzyme treatments yielded only partial improvements, confirming the necessity of synergy to fully dismantle the NSP barrier, the enzymatic optimization of texture and structure provides a viable clean-label strategy for developing nutritious and acceptable HB-enriched baked goods, despite the persistent influence of raw materials on color and aroma. These findings offer not only a practical solution for valorizing highland barley in staple foods, but also a deeper understanding of how enzyme-based physical modification can redirect complex cereal systems toward desirable structural and functional outcomes—a perspective that may extend to other coarse grains or composite flour applications.

## Figures and Tables

**Table 1:** Effects of Different Treatments on Dough Texture Characteristics.

Sample	Hardness (g)	Springiness	Chewiness (g)	Cohesiveness	Gumminess (g)
WF	85.67 ± 1.44 ^c^	0.81 ± 0.02 ^c^	57.60 ± 2.74 ^e^	0.83 ± 0.03 ^b^	71.38 ± 2.41 ^e^
HB	2591.87 ± 160.95 ^a^	0.28 ± 0.01 ^d^	259.07 ± 11.33 ^a^	0.34 ± 0.03 ^d^	855.24 ± 57.61 ^a^
40	435.98 ± 10.88 ^b^	0.32 ± 0.01 ^d^	51.16 ± 2.40 ^e^	0.37 ± 0.01 ^c^	159.83 ± 2.56 ^e^
40-X	109.89 ± 2.21 ^c^	0.87 ± 0.01 ^ab^	83.15 ± 0.72 ^c^	0.87 ± 0.01 ^a^	95.58 ± 0.75 ^c^
40-B	144.61 ± 3.17 ^c^	0.88 ± 0.01 ^a^	105.12 ± 2.09 ^b^	0.82 ± 0.01 ^b^	118.55 ± 1.76 ^b^
40-X+B	97.95 ± 4.69 ^c^	0.86 ± 0.00 ^b^	68.23 ± 3.27 ^d^	0.81 ± 0.00 ^b^	79.34 ± 3.80 ^d^

WF: completely made from high-gluten wheat flour, HB: completely made from highland barley with bran, 40: composed of 40% HB and 60% WF, 40-X: sample 40 was treated with xylanase, 40-B: sample 40 was treated with β-glucanase, 40-X+B: sample 40 was treated with β-glucanase. ^a–e^ Different letters indicate significant differences between groups (*p* < 0.05).

**Table 2:** Effects of Various Enzyme Treatments on the Texture Characteristics of Bread.

Sample	Hardness (g)	Springiness	Chewiness (g)	Cohesiveness	Gumminess (g)
WF	337.83 ± 11.94 ^c^	0.76 ± 0.05 ^a^	173.02 ± 15.08 ^c^	0.68 ± 0.02 ^a^	217.62 ± 23.41 ^b^
HB	4643.85 ± 328.93 ^a^	0.42 ± 0.06 ^c^	519.93 ± 77.18 ^a^	0.27 ± 0.05 ^d^	1274.99 ± 329.46 ^a^
40	843.06 ± 39.58 ^b^	0.74 ± 0.03 ^a^	284.35 ± 13.35 ^b^	0.46 ± 0.03 ^c^	384.65 ± 23.84 ^b^
40-X	713.98 ± 53.14 ^b^	0.73 ± 0.05 ^a^	289.39 ± 30.37 ^b^	0.56 ± 0.06 ^b^	397.62 ± 16.70 ^b^
40-B	683.55 ± 12.48 ^b^	0.70 ± 0.04 ^b^	237.43 ± 47.48 ^c^	0.49 ± 0.07 ^c^	337.67 ± 53.12 ^b^
40-X+B	644.71 ± 47.33 ^b^	0.62 ± 0.06 ^b^	180.76 ± 22.98 ^c^	0.45 ± 0.04 ^c^	293.28 ± 45.53 ^b^

WF: completely made from high-gluten wheat flour, HB: completely made from highland barley with bran, 40: composed of 40% HB and 60% WF, 40-X: sample 40 was treated with xylanase, 40-B: sample 40 was treated with β-glucanase, 40-X+B: sample 40 was treated with β-glucanase. ^a–d^ Different letters indicate significant differences between groups (*p* < 0.05).

## Data Availability

The original contributions presented in this study are included in the article. Further inquiries can be directed to the corresponding authors.

## Abbreviations

The following abbreviations are used in this manuscript:

HB	Whole grain highland barley
NSPs	Non starch polysaccharides
BGS	β-glucanase
WF	High gluten wheat flour
40	flour composed of 40% “HB” and 60% “WF”
40-X	Sample “40” was treated with xylanase.
40-B	Sample “40” was treated with β-glucanase
40-X+B	Sample “40” was treated with xylanase and β-glucanase
